# Relationship between Autonomic Nervous System Function and Continuous Interstitial Glucose Measurement in Patients with Type 2 Diabetes

**DOI:** 10.1155/2014/835392

**Published:** 2014-08-03

**Authors:** Stavroula Kalopita, Stavros Liatis, Petros Thomakos, Ioannis Vlahodimitris, Chryssoula Stathi, Nicholas Katsilambros, Nicholaos Tentolouris, Konstantinos Makrilakis

**Affiliations:** ^1^First Department of Propaedeutic Internal Medicine, Athens University Medical School, Laiko General Hospital, 17 Agiou Thoma Street, 11527 Athens, Greece; ^2^Department of Cardiology, Laiko General Hospital, 17 Agiou Thoma Street, 11527 Athens, Greece

## Abstract

*Aims*. The Aim of the present study was to examine whether there is a relationship between autonomic nervous system function and glycemic variability (GV) in patients with type 2 diabetes (T2D). *Methods*. A total of 50 (29 males) patients with T2D (mean age 58.4 ± 9.9 years, median diabetes duration 5.5 [IQR 2.0–9.25] years), on oral antidiabetic agents, underwent ECG recording and subcutaneous glucose monitoring, simultaneously and continuously, for 24 hours. *Results*. After adjustment for HbA1c and diabetes duration, total power of heart rate variability (HRV) was inversely associated with the standard deviation of the mean interstitial tissue glucose (MITG) and with the *M*-value during the entire recording (*r*: −0.29, *P* = 0.052; *r*: −0.30, *P* = 0.047, resp.) and during the night (*r*: −0.29, *P* = 0.047; *r*: −0.31, *P* = 0.03, resp.). Most of the HRV time-domain indices were significantly correlated with standard deviation of the MITG and the *M*-value. These correlations were stronger for the HRV recordings during the night. No significant association was found between HRV parameters and MAGE. *Conclusions*. HRV is inversely associated with GV in patients with T2D, which might be a sign of causation between GV and autonomic dysfunction. Prospective studies are needed to further investigate the importance of GV in the pathogenesis of long-term complications of diabetes.

## 1. Introduction

In normal individuals the heart rate has a high degree of beat-to-beat variability, fluctuating with respiration, increasing with inspiration and decreasing with expiration. Cardiac autonomic neuropathy (CAN) in diabetes results from damage to the autonomic nerve fibers (parasympathetic and sympathetic) that innervate the heart and blood vessels, with resultant abnormalities in heart rate control and vascular dynamics [1], ultimately leading to diminished heart rate variability (HRV) [2]. Clinical symptoms of autonomic dysfunction may not appear until long after diabetes onset, but subclinical CAN, manifested by reduced HRV (the earliest indicator of CAN), may be detected as early as within 1-2 years after diagnosis of diabetes [3].

CAN is an often overlooked complication of diabetes [4], associated with dire consequences regarding morbidity and mortality [5]. It carries a high risk of cardiac arrhythmias and sudden death, possibly related to silent myocardial ischemia [6]. There is no widely accepted single approach to the diagnosis of CAN in diabetes. Assessment of heart rate variability (HRV), orthostatic hypotension, and 24 h blood pressure profiles provide indices of both parasympathetic and sympathetic autonomic function and can be used in clinical settings. Specifically, HRV provides a noninvasive and objective method for assessing cardiovagal function and may be derived from electrocardiogram recordings [7]. Twenty-four-hour ECG recordings allow calculation of more complex statistical time-domain measures and together with spectral analysis of HRV allow for a better delineation of cardiac autonomic function [8].

The concept of glycemic variability (GV) in type 2 diabetes (T2D) has recently attracted great interest for several reasons, including association with higher levels of markers of oxidative stress [9] and increased mortality in certain circumstances, such as sepsis and critical illness [10]. Despite experimental evidence, however, in clinical ground, there is insufficient evidence to support an independent relationship between blood glucose fluctuations and long-term complications of diabetes; the issue remains to be controversial [11]. So far it is not known whether glycemic variability is associated with cardiac autonomic dysfunction in diabetic patients [12]. It is intriguing to hypothesize that increased glycemic variability may lead to autonomic imbalance through oxidative stress and acute elevation of proinflammatory cytokines [13]. Glycemic variability can be assessed more precisely nowadays with the use of special devices [14] that measure the interstitial tissue glucose levels continuously over a period of several days (continuous glucose monitoring systems [CGMS]) [15].

The aim of the present cross-sectional study was to investigate the relationship between cardiac autonomic function (evaluated by HRV during continuous ECG recording) and GV (assessed by simultaneous continuous interstitial tissue glucose monitoring) in patients with type 2 diabetes.

## 2. Materials and Methods

### 2.1. Study Sample and Standard Procedures

The study population included 50 (29 males) patients with T2D (mean age 58.4 ± 9.9 years, median diabetes duration 5.5 [IQR 2.0–9.25] years), treated with oral antidiabetic agents. The patients who took part in the study were attending the outpatient diabetes clinic of the Laiko General Hospital in Athens, Greece. Patients receiving insulin or medications affecting cardiac rate and patients with uncontrolled thyroid disease, alcoholism, or any acute illness were excluded. All patients had a complete screening history and physical examination before inclusion into the study, to assess for the presence of microvascular (retinopathy, nephropathy, and neuropathy) or macrovascular complications.

Participants presented to the diabetes research clinic in the morning, after an overnight fast. They had been asked to abstain from alcohol and smoking for at least 12 hours before presentation and not to take their glucose-lowering and antihypertensive therapy (if any) on the morning of each visit.

Body weight and height were measured in light clothing and body mass index (BMI, the ratio of weight [in kg] to height [in m^2^]) was calculated. Brachial blood pressure (BP) was measured using a semiautomatic BP device with the subjects rested for 5 minutes. Blood samples were obtained in order to measure fasting plasma lipids (total cholesterol, triglycerides, HDL-cholesterol, and LDL-cholesterol), as well as glucose and HbA1c. LDL-cholesterol was calculated using the Friedewald formula [16].

The study was carried out in accordance with the principles of the Declaration of Helsinki as revised in 2008 [17] and was approved by the participating hospital's ethics committee. Written informed consent was obtained from all participants.

### 2.2. Evaluation of Autonomic Function

All patients underwent continuous ECG Holter monitoring for 24 hours. For this study the digital ECG Holter recorder Spider View (ELA Medical, France) with seven electrodes was used to record three-channel ECGs. The 24-hour recordings were analysed using the SyneScope Holter analysis software (version 3.00 ELA Medical, France). Artefacts and ectopic beats were automatically excluded from analysis. In addition, the QRS complex classification was reviewed by an experienced cardiologist blinded to the patients' clinical characteristics. All of the HRV time- and frequency-domain parameters, recommended by the Task Force of the European Society of Cardiology and the North American Society of Pacing and Electrophysiology, were calculated based on the 24-hour recordings [7]. The values of time-domain parameters were expressed in milliseconds (ms). HRV in the frequency-domain was computed by SyneScope using fast Fourier transformation analysis. The total power and, respectively, the power in very low frequency (VLF, ≤0.04 Hz), low frequency (LF, 0.04–0.15 Hz), and high frequency (HF, 0.15–0.40 Hz) bands were measured. All the frequency-domain parameters of HRV were calculated as absolute values and expressed in ms^2^. Furthermore, the LF and HF powers were expressed in normalised units (nu), representing the relative value of each power component in proportion to the total power minus the VLF component [7].

### 2.3. Evaluation of Glucose Variability

A commercially available device based on the microdialysis technique was used for the continuous interstitial glucose monitoring (GlucoDay, Menarini Diagnostics). A microdialysis fiber was inserted by a medical professional and calibrated with a blood glucose test; glucose results were stored for retrospective analysis for 24 h, acquiring data every 5 min (288 measurements/day), after which the fiber was removed [14]. The CGMS was calibrated in a standard way as recommended by the manufacturer, using four validated self-monitored blood glucose levels during the 24 h monitoring period [18]. During this period, the patients were asked to have normal activities at home and at work and were advised to take their medications as usual. The following indices of glycemic variability were calculated from the stored glucose data:

- the standard deviation of the mean glucose value (SDMG), a measure of the dispersion of all the blood glucose values;
- the mean amplitude of glycemic excursions (MAGE) calculated by taking the arithmetic mean of the interstitial tissue glucose increases or decreases (from nadirs to peaks or vice versa) provided that the differences are greater than one SD of the mean glucose value [19];
- the *M*-value, a logarithmic transformation of the deviation of glycemia from an arbitrary assigned “ideal” glucose value (6.67 mmol/L [120 mg/dl]) [20].

### 2.4. Statistical Analysis

Normally distributed variables are presented as mean ± SD, while the median (25th–75th, interquartile range) is used for variables with a skewed distribution. Continuous variables were tested for normal distribution by the Kolmogorov-Smirnov test. Data not normally distributed were log-transformed for analysis. Hence, all HRV and GV indices were log-transformed due to their skewed distribution. Pearson's correlation coefficient was used to investigate associations between variables. Partial correlation was used to control for the effect of other, possibly confounding, variables. All reported *P* values are from two-sided tests and compared to a significance level of 5%. Data were analyzed using the Statistical Package SPSS, version 19.0 (SPSS Inc., Chicago, IL, USA).

## 3. Results

The demographic and clinical characteristics of the patients are shown in Table 1. They had relatively good glycemic control, as evidenced by their adequate HbA1c levels. The great majority (94%) were being treated with one or more glucose-lowering medications, mostly metformin (52%), sulphonylureas, (20%), and sitagliptin (16%), in various combinations. The mean BMI was 30.8 ± 5.7 kg/m². More than half of the patients (62%) had been diagnosed with arterial hypertension and 42% were smokers.

The glycemic indices derived from CGMS are presented in Table 2. Mean interstitial tissue glucose (MITG) had a positive correlation with HbA1c (*r*: 0.55, *P* < 0.001), while the GV indices were also significantly (but weakly) positively correlated with HbA1c (MAGE: *r*: 0.32, *P* = 0.02; SDMG: *r*: 0.31, *P* = 0.03; *M*-value: *r*: 0.27, *P* = 0.059) and with diabetes duration (MAGE: *r*: 0.32 *P* = 0.02; SDMG: *r*: 0.36, *P* = 0.01; *M*-value: *r*: 0.29, *P* = 0.04).

Total power of HRV was negatively correlated with HbA1c (*r*: −0.32, *P* = 0.024) but not with age, BMI, diabetes duration, systolic and diastolic BP, smoking, or lipid parameters. The correlation with HbA1c was stronger during the day (*r*: −0.38, *P* = 0.009) than during the night (*r*: −0.31, *P* = 0.03). Of the other HRV frequency-domain parameters, HF, but not LF, was correlated with HbA1c in a similar pattern as total power, while the ratio of LF/HF was positively correlated with HbA1c (*r*: 0.29, *P* = 0.04). Concerning the time-domain parameters of HRV, HbA1c was significantly correlated with SDNN and RMSSD, only during the night (*r*: −0.32, *P* = 0.02 and *r*: −0.36, *P* = 0.02, resp.). Total power of HRV was inversely and significantly associated with MITG (*r*: −0.36, *P* = 0.01). The other parameters of frequency-domain analysis were not significantly related to MITG. Most time domain parameters of HRV were inversely associated with MITG, but this correlation concerns only HRV parameters at night period. All associations, however, between HRV parameters and MITG disappeared after adjustment for HbA1c and duration of diabetes (Tables 3 and 4).

There was an overall inverse correlation between both frequency- and time-domain parameters of HRV and indices of GV. In univariable analysis, the correlation between total power and SDMG was significant (*r*: −0.30, *P* = 0.03) and stronger for the night component (*r*: −0.32, *P* = 0.02). Total power was also significantly correlated with *M*-value (*r*: −0.34, *P* = 0.02 for the total time recording and *r*: −0.36, *P* = 0.01 for the night component). The remaining parameters of frequency-domain analysis (LF, HF and LF/HF) were not significantly correlated with GV, while the time-domain indices (SDNN, PNN30, PNN50 and SDANN) were significantly correlated with SDMG and *M*-value. The above significant correlations were stronger for the night component of all recordings. There was no statistically significant association between MAGE and indices of HRV. The relation between HRV parameters and GV was further evaluated after adjustment for HbA1c and diabetes duration, resulting in slight attenuation but remaining, in most cases, statistically significant (Tables 3 and 4).

## 4. Discussion

In the present study, it was shown for the first time that GV, calculated during a 24 h CGMS recording, is independently associated with autonomic nervous system function, assessed by simultaneous analysis of HRV, in patients with type 2 diabetes. To our knowledge, only one previous study has investigated HRV and GV in parallel, by simultaneous ECG and CGMS recordings, in patients with type 2 diabetes [21]. However, in that study, only a positive association (*r*: 0.40, *P* = 0.04) between the ratio of LF/HF and MAGE was reported.

The association between HRV and glycemia in the present study has been observed at three levels. At the first level, total power, a measure of total variance of HRV, and HF, a measure of vagal activity [7], were inversely associated with HbA1c, a measure of average glycemia during the three months preceding the experiment. At the second level, total power was inversely associated with MITG, a measure of glycemic exposure during the experiment. Interestingly, MITG was also inversely associated with SDNN and SDANN, both indices of overall variability in time-domain analysis of HRV recordings [7]. At the third level, it was shown that overall heart rate variance, expressed by the total power in frequency-domain analysis and by SDNN, PNN50, and SDANN in time-domain analysis, was inversely, significantly, and independently associated with GV, expressed by *M*-value and (partly) by SDMG, but not by MAGE.

The inverse association between HRV and chronic hyperglycemia has been previously shown in several studies in patients with and without diabetes and there is strong evidence suggesting that chronic hyperglycemia is involved in the development of autonomic neural imbalance [2, 22, 23]. The presence of reduced HRV in patients with diabetes has been attributed to cardiac autonomic impairment, which appears to be present at early stages of diabetic metabolic impairment [23, 24]. The association, however, between HRV and simultaneously/continuously recorded glycemia has been investigated to a much lesser extent. To our knowledge, in the only previous published study where simultaneous HRV and glucose readings were examined, the relation between HRV indices and mean glucose levels was not reported [21]. In the present study, indices of HRV were inversely related with MITG, but the associations were not statistically significant after adjustment for HbA1c and duration of diabetes. On the contrary, the association between certain indices of HRV (total power, SDANN, SDNN, and PNN50) and GV were sustained after such adjustment (Tables 3 and 4).

The association between GV and autonomic function in the present study should be interpreted with caution. The cross-sectional design does not allow for causal relationships to be inferred. A possible association between GV and autonomic dysfunction has been suggested on the basis of a hypothesized broader association between GV and the development of chronic diabetic complications. Although the role of chronic hyperglycemia in the development of small/large vessel and nerve damage is well established [25], whether or not GV is independently involved in the pathogenesis of microvascular and macrovascular diabetic complications is an issue of debate [11]. In a separate analysis of DCCT data, Kilpatrick et al. [26] showed that the variability in blood glucose around a patient's mean value has no influence on the development or progression of either retinopathy or nephropathy in type 1 diabetes. Another analysis of the DCCT data [27], however, showed that increasing variability in HbA1c adds to the risk of microvascular complications, implying a significant role of longer-term glucose variability in the development of retinopathy and nephropathy in type 1 diabetes, in contrast to the effect of short-term glucose variability on complication risk.

The inverse HRV/GV association in the present study could also be attributed to a short-term influence of acute glucose elevations on sympathetic activity. It has been repeatedly shown that both hyperglycemia and hyperinsulinemia produce sympathetic activation in normal individuals [28], while the effect of glucose peaks on sympathetic function in patients with diabetes is not known. On the other hand, it is well known that hypoglycemia stimulates an acute adrenergic response both in normal individuals and in patients with diabetes [29]. Furthermore, this association might be looked at from the opposite angle. It might be thus hypothesized that autonomic nervous system dysfunction could result in larger fluctuations in blood glucose, due to disturbed gastrointestinal tract motility or an impaired counterregulatory response to low glycemic levels [30]. However, the short duration of diabetes, the absence of other complications, and the relatively high mean HRV indices of the study population render this possibility rather unlikely.

The association between HRV and GV was observed only for certain indices. Hence, in frequency-domain analysis, only total power was significantly related to GV, while most time-domain indices had a significant association with GV, especially during the night. It should be noted that the interpretation of frequency-domain analysis calculations during long-term recordings is not well defined [7]. Physiological mechanisms of heart period modulations are not stationary during the 24-hour period; hence the physiological interpretation of the spectral components (VLF, LF, and HF power components) calculated over 24 hours is difficult. On the other hand, time-domain indices are more reliable measures of overall heart rate variance on the long term [7].

The GV indices associated with HRV included SD of mean glucose and *M*-value, while no association was observed between MAGE and HRV. Although there is no “gold standard” for determining glucose variability, according to a recent review [31] SDMG might be the preferable method when quantifying variability from CGMS data, because it is the easiest and best validated measure. In the same review it is suggested that *M*-value is rather a clinical than a mathematical indicator of glycemic control, because hypoglycemia has a greater impact on it than hyperglycemia [31]. Furthermore, MAGE arbitrarily ignores glucose excursions of less than 1 SD [19], a fact that may incorrectly disregard possibly important smaller excursions [31]. As the population of the present study had a relatively short diabetes duration and good glycemic control, it is possible that glycemic fluctuations were not sufficiently captured by MAGE.

The main strength of the present study is that the investigation of HRV and GV has been performed simultaneously during 24 hours. Furthermore, the study population consisted of patients with early-stage type 2 diabetes and good glycemic control, allowing for detection of early disturbances in HRV with relatively modest fluctuations in glucose levels. In addition, the latter was measured with CGMS, in order to capture variations in interstitial tissue glucose levels as frequently as every 5 minutes.

The main limitation of the study is that its cross-sectional design does not allow for causal relationships to be identified. Hence, the observed associations may only serve as hypothesis generating.

In conclusion, the present study showed that HRV is associated with GV in patients with type 2 diabetes with a relatively short course of disease. This association might be a sign of causation between GV and diabetic complications, although other explanations could also apply. Prospective studies are needed to further investigate the importance of GV in the pathogenesis of autonomic dysfunction and other long-term complications of diabetes.

## Figures and Tables

**Table 1:** Patients' demographic and clinical characteristics.

Variable	Value
*N *	50
Gender [male *n* (%)]	29 (58)
Age (years)	58.4 ± 9.9
BMI (kg/m²)	30.8 ± 5.7
Systolic blood pressure (mm Hg)	128.3 ± 15.5
Diastolic blood pressure (mm Hg)	74.9 ± 10.2
Fasting glucose (mmol/L)	7.7 ± 2.2
Total cholesterol (mmol/L)	4.7 ± 0.9
Triglycerides (mmol/L)∗	1.6 (1.2–2.2)
HDL-C (mmol/L)	1.2 ± 0.2
LDL-C (mmol/L)	2.8 ± 0.9
Fasting glucose (mmol/L)	7.7 ± 2.2
HbA1c (mmol/mol)	54.3 ± 12.6
Duration of diabetes (years)∗	5.5 (2.0–9.25)
Presence of hypertension	31 (62)
Smokers	21 (42)
Treatment with sulphonylureas	26 (52)
Treatment with metformin	47 (94)
Treatment with meglitinides	1 (2)
Treatment with thiazolidinediones	8 (16)
Treatment with sitagliptin	10 (20)

(Data are presented as mean (±SD) or as *n* (%).

∗Median value (interquartile range).

BMI: body mass index, HDL-C: high density lipoprotein cholesterol, and LDL-C: low density lipoprotein cholesterol.

Hypertension was defined as systolic blood pressure >140 mm Hg or diastolic blood pressure >90 mm Hg or the use of antihypertensive medication.

**Table 2:** Indices related to glycemia as obtained from the continuous glucose monitoring system (CGMS).

Variable	Value
Mean interstitial tissue glucose (mmol/L)	7.9 ± 1.7
SD of mean glucose∗ (mmol/L)	2.13 (1.76–0.05)
*M*-value∗	55.1 (37.67–119.85)
MAGE∗	92.0 (75.20–121.20)

Data are expressed as mean ± SD.

∗Median value (interquartile range).

MAGE: mean amplitude of glycemic excursions.

**Table 3:** Correlation analysis between frequency-domain parameters of HRV, mean interstitial tissue glucose, and indices of glycemic variability.

Variable	Mean interstitial tissue glucose		MAGE		SD of mean glucose		*M*-value	
	*r*	*P*	*r*	*P*	*r*	*P*	*r*	*P*
TP (t)	−0.25	0.09	−0.14	0.37	**−0.29**	**0.052**	**−0.30**	**0.047**
TP (n)	−0.25	0.09	−0.13	0.39	**−0.29**	**0.047**	**−0.31**	**0.034**
LF (t)	−0.19	0.22	−0.03	0.82	−0.14	0.35	−0.13	0.38
LF (n)	−0.20	0.19	−0.02	0.90	−0.14	0.36	−0.14	0.35
HF (t)	−0.08	0.59	0.05	0.76	−0.10	0.51	−0.16	0.29
HF (n)	−0.09	0.57	0.07	0.63	−0.09	0.56	−0.16	0.28
LF/HF (t)	−0.13	0.37	−0.11	0.46	−0.05	0.76	0.05	0.73
LF/HF (n)	−0.11	0.48	−0.12	0.44	−0.04	0.81	0.07	0.67

Data are adjusted for HbA1c and duration of diabetes.

All variables are log-transformed due to skewness.

(t): Total; (n): night

Night period: 23:00–06:00

TP: Variance of all normal-to-normal NN intervals

LF: Power in the low frequency range

HF: Power in the high frequency range

LF/HF: Ratio LF/HF.

**Table 4:** Correlation analysis between time-domain parameters of HRV, mean interstitial tissue glucose, and indices of glycemic variability.

Variable	Mean interstitial tissue glucose		MAGE		SD of mean glucose		*M*-value	
	*r*	*P*	*r*	*P*	*r*	*P*	*r*	*P*
SDRR (t)	−0.29	0.052	−0.06	0.70	−0.23	0.13	**−0.28**	**0.06**
SDRR (n)	−0.28	0.058	−0.19	0.20	**−0.39**	**0.007**	**−0.40**	**0.006**
PNN30 (t)	−0.07	0.64	−0.02	0.87	−0.15	0.32	−0.25	0.09
PNN30 (n)	−0.14	0.36	−0.06	0.72	−0.19	0.22	−0.27	0.07
PNN50 (t)	−0.11	0.47	−0.06	0.72	−0.14	0.36	−0.27	0.07
PNN50 (n)	−0.20	0.19	−0.10	0.49	**−0.23**	**0.12**	**−0.35**	**0.02**
SDANN (t)	−0.29	0.054	−0.04	0.79	−0.21	0.16	−0.27	0.07
SDANN (n)	−0.25	0.09	−0.18	0.22	**−0.38**	**0.009**	**−0.37**	**0.01**
RMMSD (t)	0.05	0.75	0.03	0.86	−0.04	0.80	−0.14	0.36
RMSSD (n)	−0.01	0.93	0.02	0.87	−0.08	0.62	−0.18	0.24

Data are adjusted for HbA1c and duration of diabetes.

All variables are log-transformed due to skewness.

(t): Total; (n): night.

Night period: 23:00–06:00

SDRR: Standard deviation of all normal-to-normal RR intervals

PNN30: Number of pairs of adjacent NN intervals differing >30 ms in the entire recording divided by the total number of all NN intervals

PNN50: Number of pairs of adjacent NN intervals differing >50 ms in the entire recording divided by the total number of all NN intervals

SDANN: 5 min, standard deviation of the changes of NN intervals in all 5-minute segments of the entire recording

RMSSD: The square root of the mean of the sum of the squares of differences between adjacent NN intervals.
